# Anti-Influenza Effect of Nanosilver in a Mouse Model

**DOI:** 10.3390/vaccines8040679

**Published:** 2020-11-13

**Authors:** Irina V. Kiseleva, Mohammad Al Farroukh, Ekaterina A. Skomorokhova, Andrei R. Rekstin, Ekaterina A. Bazhenova, Daria N. Magazenkova, Iurii A. Orlov, Larisa G. Rudenko, Massimo Broggini, Ludmila V. Puchkova

**Affiliations:** 1 Federal State Budgetary Scientific Institution “Institute of Experimental Medicine”, 197376 Saint Petersburg, Russia; mouhammad1farroukh@gmail.com (M.A.F.); ekaterina-skomorohova@mail.ru (E.A.S.); arekstin@yandex.ru (A.R.R.); sonya.01.08@mail.ru (E.A.B.); magdash@mail.ru (D.N.M.); vaccine@mail.ru (L.G.R.); 2 International Research Center of Functional Materials and Devices of Optoelectronics, ITMO University, 197101 Saint Petersburg, Russia; orlov239@gmail.com; 3 Laboratory of Molecular Pharmacology, Istituto di Ricerche Farmacologiche “Mario Negri” IRCCS, 20156 Milan, Italy; massimo.broggini@marionegri.it

**Keywords:** ceruloplasmin, copper status, silver nanoparticles, influenza, influenza virus replication, prophylaxis and treatment, indirectly-acting antiviral drug

## Abstract

The present study assesses copper metabolism of the host organism as a target of antiviral strategy, basing on the “virocell” concept. Silver nanoparticles (AgNPs) were used as a specific active agent because they reduce the level of holo-ceruloplasmin, the main extracellular cuproenzyme. The mouse model of influenza virus A infection was used with two doses: 1 LD_50_ and 10 LD_50_. Three treatment regimens were used: Scheme 1—mice were pretreated 4 days before infection and then every day during infection development; Scheme 2—mice were pretreated four days before infection and on the day of virus infection; Scheme 3—virus infection and AgNP treatment started simultaneously, and mice were injected with AgNPs until the end of the experiment. The mice treated by Scheme 1 demonstrated significantly lower mortality, the protection index reached 60–70% at the end of the experiment, and mean lifespan was prolonged. In addition, the treatment of the animals with AgNPs resulted in normalization of the weight dynamics. Despite the amelioration of the infection, AgNP treatment did not influence influenza virus replication. The possibility of using nanosilver as an effective indirectly-acting antiviral drug is discussed.

## 1. Introduction

Influenza annually places a tremendous burden on the global health system and has a significant economic impact. Vaccination is the most effective way to prevent influenza. The Centers for Disease Control and Prevention, the World Health Organization, the US National Vaccine Advisory Committee, and other health care and public health agencies and professional organizations consider vaccination as the most effective method for preventing influenza infection and possible hospitalization or death [1,2,3]. Many years after the initial release of the influenza vaccination, its benefits for all age groups, particularly children, remain unquestioned [4].

Another important measure is specific anti-influenza chemotherapy, for which amantadine-type drugs or neuraminidase inhibitors are used. These measures aim at reducing the incidence of influenza and prevention of the development of its severe forms and complications [5]. The success of both approaches is limited by high genetic variability of the influenza virus, which allows the virus to rapidly gain resistance to chemotherapeutic agents. The recurring antigenic shifts and introduction of new antigenic variants of the influenza virus into the human population greatly reduce the efficiency of vaccination. In this work, an alternative approach is followed. It is based on the “virocell” [6,7,8] and systems biology concepts [9,10,11]. These concepts suggest that the targets for suppressing viral reproduction can be found in the host’s metabolism. The present article assesses copper metabolism as a target of antiviral strategy.

The following considerations were taken into account to make this choice. First, copper, being an essential micronutrient, takes part in numerous basic functions in the cell and throughout the body. It is a factor that regulates signaling [12,13,14] and apoptosis [15]; controls the ratio of glycolysis and oxidative phosphorylation through copper-dependent transcription factors [16,17,18], neovascularization [19], neurotransmission [20], proliferation [21]; and is a co-activator of odorant receptors [22]. Furthermore, copper serves as a catalytic and structural co-factor of vital enzymes that control tissue respiration (cytochrome-c-oxidase, COX), antioxidant protection (Cu/Zn-superoxide dismutases, SOD1 and SOD3), the formation of connective tissue (lysyl oxidases), iron redox reactions Fe(2+) → Fe(3+) (blue multicopper oxidases), processing of neuropeptides (peptidyl-glycine alpha-amidating monooxygenase), and formation and degradation of the neurotransmitters (dopamine-β-hydroxylase and neurospecific copper oxidases) [23].

Second, copper metabolism was chosen because the processing of the influenza virus genetic program is closely related to changes in copper metabolism of the host cell. Thus, in the early stages of virus reproduction, virus-induced disruption of cytosolic Cu/Zn-SOD formation [24,25,26,27] and antagonism to copper-mediated host innate immune responses are observed [28]. The effect of the influenza virus on host copper metabolism also manifests itself in loss of smell [29]; this is also true for COVID-19 [30]. In addition, the virus does not multiply in cells with knockdown genes providing copper import into the cell and its transfer to the Golgi complex lumen [31]. Copper in the form of Cu(2+) is required for RNA-dependent RNA polymerase activity of influenza viruses, and for maintaining the structure and functions of viruses [32,33]. Moreover, the M2 membrane protein of the influenza virus forms a proton-selective ion channel that binds copper ions with high affinity, and is possibly regulated by copper [34]. Additionally, ceruloplasmin (Cp), a major copper-containing extracellular N-glycoprotein, affects the reproduction of the influenza virus in vitro and in vivo in both the early and late stages of multiplication. These experimental data revealed that the action of Cp was realized via its enzymatic activity and the glycan moiety [35,36,37]. Serum Cp is a member of the multi-copper blue oxidase family, which also includes hephaestin, zyklopen, and GPI-Cp, bound to the plasma membrane through the glycosyl phosphatidylinositol anchor [38]. Cp belongs to the “moonlighting” category of proteins, that is, proteins that display more than one physiologically relevant biochemical or biophysical function within one polypeptide chain [39]. It also belongs to the group of acute-phase proteins. It is the main ferroxidase and extracellular antioxidant copper donor to cells of non-hepatic lineages, and it is involved in the regulation of neutrophil apoptosis [40,41,42]. The Cp level in blood serum is the main biomarker of copper status, the indicators of which (copper status indexes: Cp oxidase activity, Cp protein concentration, and atomic copper concentration) characterize the balance of copper in the organism [43].

Finally, copper status indexes can be altered experimentally. Thus, Cp oxidase activity can be relatively quickly decreased in vivo by treatment with Ag(1+) ions and returned to its normal value by canceling the treatment [44]. This is possible due to the similarity of valence shells of Ag(1+) and Cu(1+), thus Ag(1+) ions are mistakenly bound by copper transporters and passed through copper extracellular and intracellular communication pathways [45]. However, the indexes also quickly return to their normal values by canceling silver ion treatment; since silver, like copper, is rapidly excreted from the body. Silver nanoparticles (AgNPs) have the same mode of action as silver ions because, in organisms, Ag(1+) ions are released from nanoparticles in the process of chemical modification [46,47]. Silver from the corroding nanoparticles is delivered to the liver; in hepatocytes, it is transferred to the Golgi complex lumen and it is included in the apo-Cp that forms. Cp partially metallated with silver atoms (Ag–Cp) is characterized by a low content of copper atoms and lacks oxidase activity. In addition, nanosilver is not incorporated into the active centers of SOD1 and COX, which are vital cytosolic cuproenzymes [45,48,49]. Therefore, treatment with AgNPs cannot cause side effects due to the effect on the antioxidant system and oxidative phosphorylation [45,48,49]. At the same time, the mechanism of interaction of AgNPs with viruses is a largely unexplored field [50,51]. Concerning influenza, experiments with AgNPs are mainly limited to in vitro studies [52,53,54], and the publications describing the anti-influenza effect of AgNPs in vivo are limited [55].

The aim of this study was the evaluation of the effect of the low copper status indexes on the influenza virus infection in a mouse model. The oxidase activity in the blood of mice was experimentally reduced via intraperitoneal injection of AgNPs.

## 2. Materials and Methods

**Virus.** A/South Africa/3626/2013 (H1N1)pdm09 (SA) was obtained from The Francis Crick Institute (London, UK). The 50% lethal dose (LD_50_) of A/South Africa/3626/2013 (H1N1)pdm09 influenza virus in mice was evaluated as previously described [56] and calculated by the routine Reed and Muench method [57].

**Mice.** Female CBA mice, age of 6–8 weeks, body mass of 16–20 g, were purchased from the laboratory breeding nursery “Rappolovo” (St Petersburg Region, Russia). The animals were maintained in polycarbonate cages with wood shavings in a temperature-controlled facility (23–25 °C), with a 12:12 h light-dark cycle, 60% humidity, with standard food and water ad libitum.

**Ethics statement.** Mice were handled following European Union legislation [58] and the Russian Manual for laboratory animals [59]. The study on mice was conducted using protocol No. 1/20 approved on 27 February 2020 by the Institutional Local Ethical Committee (IEM, St. Petersburg, Russia). At the end of the study, the animals were humanely euthanized.

**Fabrication of AgNPs and their physicochemical characterization.** AgNPs were synthesized by the reduction of silver nitrate with hydrazine hydrate in the presence of potassium oleate as a stabilizer. The reagents were purchased from “Reakhim” (Moscow Region, Russia). AgNPs were characterized by UV/vis absorption with a Shimadzu UV 1800 (Shimadzu, Kyoto, Japan). The structural analysis was performed by transmission electron microscopy (TEM) using a Jeol JEM-2100F microscope (Jeol, Tokyo, Japan) at accelerating voltage of 200 kV with point-to-point resolution of 0.19 nm. Silver concentration was measured by atomic absorption spectrometry, AAS (ZEEnit 650P spectrometer, Analytik Jena, Germany). AgNP suspensions were shown to be stable during a year of storage: their color did not change, and no agglomeration was observed.

**Figure 1:**
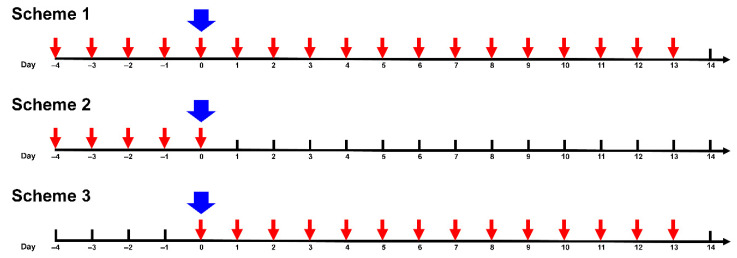
Schedules of treatment of mice with silver nanoparticles (AgNPs). Red arrow—intraperitoneal administration of AgNPs. Blue arrow—inoculation of the virus.

**Treatment and virus inoculation.** Mice were treated with AgNPs according to three Schemes: Scheme 1—mice were pretreated 4 days before infection, on the day of infection with SA influenza virus (day 0), and during a 13 day period post-infection; Scheme 2—mice were pretreated 4 days before infection with SA influenza virus and on day 0; Scheme 3—mice were treated on day 0 and during a 13 day period post-infection (Figure 1). AgNPs were injected intraperitoneally at a dose of 0.2 mg of atomic silver per 1 kg body mass daily following the schedules shown in Figure 1.

**Figure 2:**
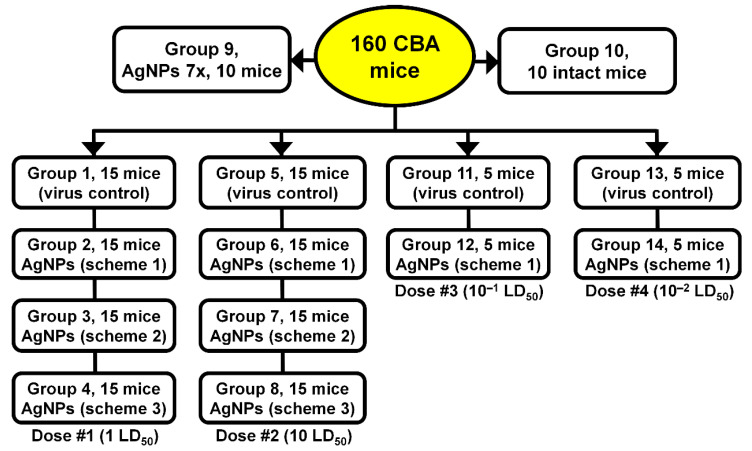
Test groups of mice.

Mice were randomly divided into eight groups, with 15 mice in each group. These groups comprised: virus control groups (1,5); groups treated by AgNPs (Ag-mice) with Scheme 1 (2,6); groups treated by AgNPs with Scheme 2 (3,7); and groups treated with AgNPs by Scheme 3 (4,8). Mice of groups 1 and 5 received virus only. Two study groups of 10 mice in each (group 9 and group 10) were used as controls. Mice of group 9 received no virus but seven injections of AgNPs daily. Mice treated with AgNPs, groups 2–4 and 6–8, were inoculated on day 0 with the virus; they were lightly anesthetized with ether and inoculated intranasally with 50 μL of PBS containing 1 (groups 2–4) or 10 (groups 6–8) LD_50_ of SA, divided equally between the nostrils. Sera for determining oxidase activity were taken after 4 (5 mice) and 7 (5 mice) injections of nanosilver. Mice in group 10 did not receive either virus or AgNPs (intact control animals) (Figure 2).

Mice were observed daily for body weight loss and lethality up to 14 days post-infection with 1 LD_50_ and 10 LD_50_ of lethal SA virus, which were the endpoint of our experiment. Weight loss of less than 10% compared to initial weight was needed before the mice were culled.

For a more comprehensive understanding of potential effects of AgNPs on virus replication, four additional groups infected with lower doses of influenza virus were added (5 mice in each group). Mice of groups 12 and 14 were treated with AgNPs according to Scheme 1. Mice of groups 11 and 12 were inoculated with 10^−1^ LD_50_ of the virus, and mice of groups 13 and 14 were inoculated with 10^−2^ LD_50_ (Figure 2).

The protection index (ratio of mortality in the control group over mortality in the experimental group) and the average life expectancy (mean days of death) were calculated according to the following formulas.

(1) $$\mathrm{Protection}\;\mathrm{index}=\frac{\mathrm{Mc}\;\mathrm{Mt}}{\mathrm{Mc}}\;\times \;100$$

where Mc and Mt are mortality (%) in the control and AgNP-treated groups, respectively.

(2) $$\mathrm{Average}\;\mathrm{life}\;\mathrm{expectancy}=\frac{\sum_{1}^{n}Di}{n}$$

where n is the number of mice in the group, i is the mouse index in the group, and Di is the number of days that mouse i lived.

**Figure 3:**
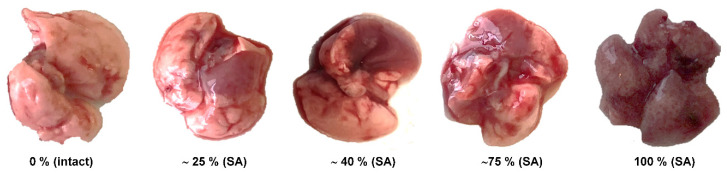
The percentage of lung area affected in mice inoculated with A/South Africa/3626/2013 (H1N1) pdm09 (SA).

**Gross pathology.** A complete macroscopic examination was performed on day 3 post-infection on animals of all groups (five mice in each group). All lung lobes were inspected. Macroscopic changes in the lungs were visually assessed as a percentage by the severity of the lesions. An example of the evaluation of macroscopic changes in the lungs can be found in Figure 3.

**Viral replication in lung tissue.** On day 3 post-infection, five mice from each of the groups infected with SA virus were sacrificed, and lungs were isolated and used for the measurement of virus load. Tissue homogenates were prepared using a small bead mill TissueLyser LT (QIAGEN, Hilden, Germany) in 1.0 mL of PBS containing antibiotic-antimycotic (Gibco, Grand Island, NY, US); the cleared supernatants were titrated in chicken embryos at the temperature of 32 °C. The lung virus titer was expressed as log_10_ EID_50_/mL/g tissue.

**Blood serum oxidase activity** was detected using the assay-in-gel method. Blood serum aliquots (1 μL) were fractioned in non-denaturing 8% polyacrylamide gel electrophoresis. Gels were stained with *o*-dianisidine to reveal the Cp oxidase activity [60]. Densitometric relative quantitation analysis of the stained gels was performed using Image Studio Lite (LI-COR Biosciences). The results were expressed as arbitrary units.

**Metal concentrations** were measured using graphite furnace atomic absorption spectrometry (AAS) with a Zeeman correction of nonselective absorption using a ZEEnit 650P spectrometer (Analytic Jena, Germany). Tissue samples for AAS were dissolved in pure HNO_3_.

**The statistical analysis** was carried out using GraphPad Prizm 7 (GraphPad Software Inc., San Diego, CA, US). A *p*-value < 0.05 was considered statistically significant.

## 3. Results

**Figure 4:**
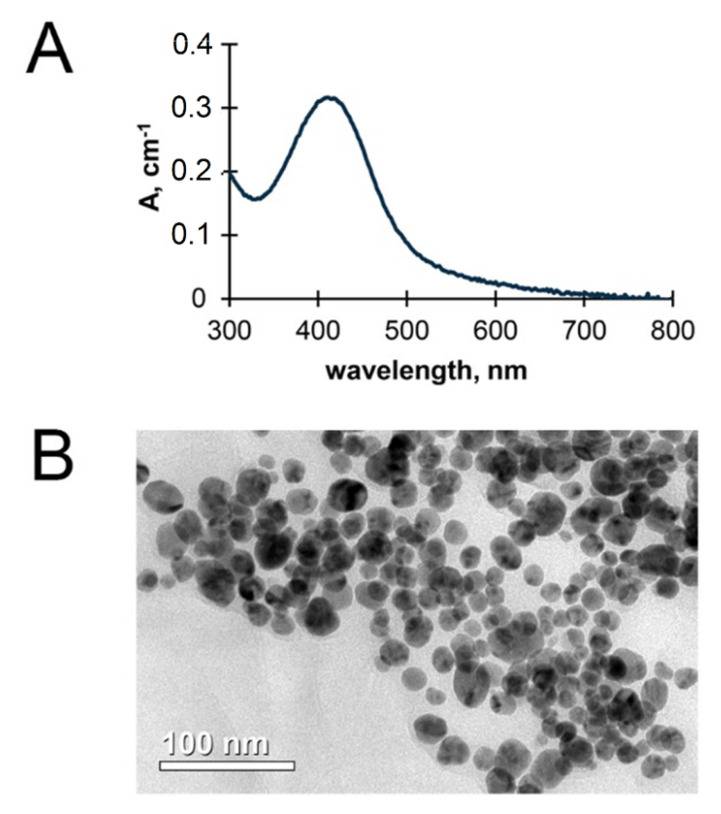
The physical properties of fabricated AgNPs: UV/Vis spectrum (**A**) and transmission electron microscopy image (**B**).

**Characterization of AgNPs used in the experiments.** The fabricated AgNPs displayed a single roughly symmetric absorption band in the UV/vis spectrum (Figure 4A), corresponding to surface plasmon excitation of nanocrystals of metallic silver. The average size of AgNPs and their shape were evaluated by TEM (Figure 4B). The fabricated AgNPs had a spherical shape. The predominant fraction of AgNPs in the sample corresponded to a diameter range of 20–25 nm (Figure 4). AgNP suspensions were shown to be stable during a year of storage, their color did not change, and no agglomeration was observed.

**Figure 5:**
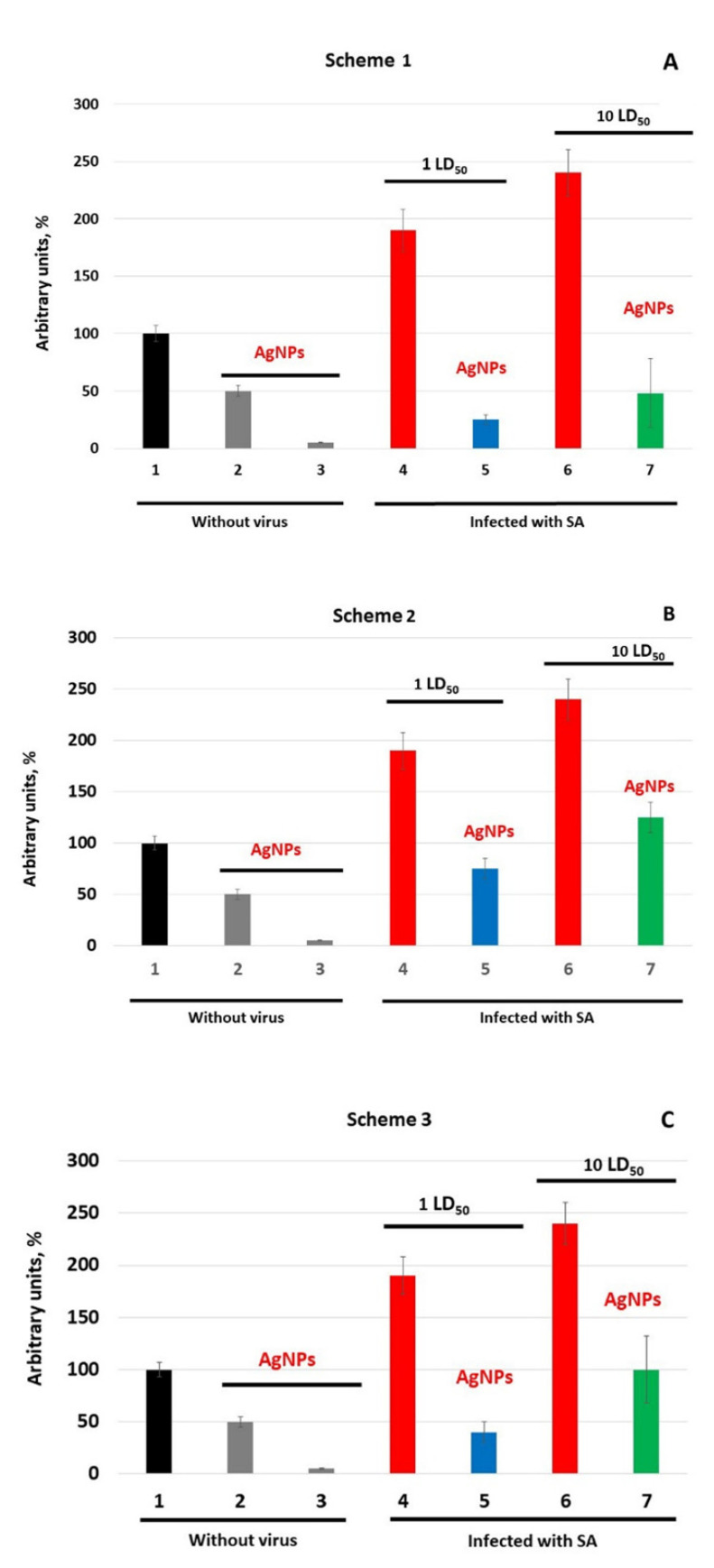
Oxidase activity of blood serum of mice against the background of influenza infection. (**A**–**C**)—mice were treated with AgNPs according to Schemes 1–3, correspondingly. Abscissa, mice groups: 1—without treatment; 2—treatment with AgNPs for 4 days; 3—treatment with AgNPs for 7 days; 4—infected with 1 LD_50_; 5—treatment with AgNPs + 1 LD_50_; 6—infected with 10 LD_50_; 7—treatment with AgNPs + 10 LD_50._

**Changes in blood oxidase activity in AgNP-treated mice after infection with influenza virus.** Three days after infection with the influenza virus, in mice that did not receive AgNPs, the oxidase activity increased approximately by a factor of 2; the effect of the increase in infecting dose was visible, but quite small (Figure 5, bar 1 versus bars 4 and 6, *p* < 0.05). These data were used as a reference for the results obtained with different AgNP treatment regimens.

In Ag-mice, the oxidase activity was lower than in the infected mice without AgNP treatment (Figure 5A–C, bars 2 and 3 versus bars 4 and 6, *p* < 0.05). In mice of group 9 treated with AgNPs, the oxidase activity in the blood decreased by a factor of 2 in 4 days and dropped to the detection limit on the 7th day of injections (Figure 5A–C, bar 1 versus bars 2 and 3). These mice served as reference groups for all groups treated by AgNPs according to different Schemes and infected with different doses of the virus.

In mice treated by AgNPs according to Scheme 1, at day 3 post-infection, oxidase activity increased by factors of 5 and 10 for 1 and 10 LD_50_ virus doses, respectively, compared with uninfected mice treated with AgNPs for 7 days (Figure 5A, bar 3 versus 5 and 7). However, the oxidase activity in Ag-mice was significantly lower than in the mice infected with the influenza virus only (Figure 5, bars 4 and 6 versus bars 5 and 7, *p* < 0.05).

In mice that did not receive AgNPs after virus infection (Scheme 2), the oxidase activity at day 3 post-infection increased by 25% at 1 LD_50_ and by 125% at 10 LD_50_, compared with uninfected mice treated with AgNPs (Figure 5B, bar 2 versus bars 5 and 7, *p* < 0.05). When AgNP treatment was started simultaneously with the influenza virus infection and continued until the end of the experiment (Scheme 3), the oxidase activity in mice was higher than in mice treated according to Scheme 1, but lower than in mice infected according to Scheme 2 (Figure 5C, bar 2 versus bars 5 (*p* > 0.05) and 7, *p* < 0.05).

In our work, another biomarker of copper status—the atomic copper concentration—was also measured. It should be borne in mind that the copper concentration and oxidase activity are not absolutely correlated because oxidase activity belongs to Cp only, however, copper is associated with Cp, albumin, and alpha-2-macroglobulin, and also found in low molecular weight substances. In mice, Cp includes about 60% of the total amount of copper [61] versus 95% in humans and rats [62]. In addition, in the sera of mice treated with AgNPs, atomic silver is included in the Cp and bound with alfa-2-macroglobulin. The obtained data are summarized in Table 1, and show that, considering all of the reservations outlined above, the data are consistent with the results presented in Figure 5.

Thus, the treatment of mice with AgNPs leads to a decrease in copper concentration. Copper content is inversely proportional to processing time. In contrast, the silver concentration increases with increased processing time. In the control group of mice infected with the influenza virus, the concentration of copper increases by about 1.5 times. AgNP treatment results in a decrease in copper concentration and an increase in silver content. These changes depend on the applied AgNP treatment Scheme. Thus, AgNPs, regardless of the infection dose, prevent the dramatic increase in oxidase activity caused by the virus. The intensity of the inhibition oxidase activity in infected mice depends on the treatment Scheme and the dose of infection. The most effective treatment is that according to Scheme 1.

**Average survival time and mortality after SA virus inoculation.** The average life expectancy (ALE, the average survival time) was calculated following inoculation with two different doses of SA virus, 1 and 10 LD_50_. The highest mortality rates were observed in control mice and mice treated by AgNPs according to Scheme 3 (Table 2).

It can be noted from Table 2 that treated animals died less rapidly than the controls. Moreover, mice infected with 1 or 10 LD_50_ of SA virus and that received AgNPs according to Scheme 1 lived longer than those that received AgNPs according to Schemes 2 and 3 (Table 2). The average survival rates of mice continuously treated by AgNPs throughout the experiment (Scheme 1) were 80% (1 LD_50_) and 60% (10 LD_50_). No mice survived the 10 LD_50_ of SA in the control (untreated) group. In contrast, 60% of mice treated according to Scheme 1 and inoculated with 10 LD_50_ survived to the end of the experiment. Amelioration of SA virus infection by silver nanoparticles was the most pronounced when mice were infected with 1 LD_50_; the mortality rate in the control group was 70%, compared to only 20% in the mice treated by AgNPs according to Scheme 1 (Table 2).

Thus, the mortality rate and survival time of mice treated by AgNPs was related to the Scheme of AgNP administration and virus dose.

**Gross pathology.** Administration of the SA virus caused significant gross morphological changes in the lungs of animals of control groups. In contrast, the histopathology results from samples of organs taken from the infected mice treated with AgNPs were closer to those obtained from the control groups 9–10 (data not shown). A minimal number of lung lesions was observed in the groups treated according to Scheme 1.

Taking together the mortality level, the average survival time, and the results of the gross pathological examination, Scheme 1 was found to be the most promising from the anti-influenza activity perspective. The most pronounced effects of AgNPs were observed in the groups treated according to this Scheme.

In further analysis, we focused on the efficacy of Scheme 1 for the prevention of influenza infection caused by 1 and 10 LD_50_ doses of the virus in more detail.

**Figure 6:**
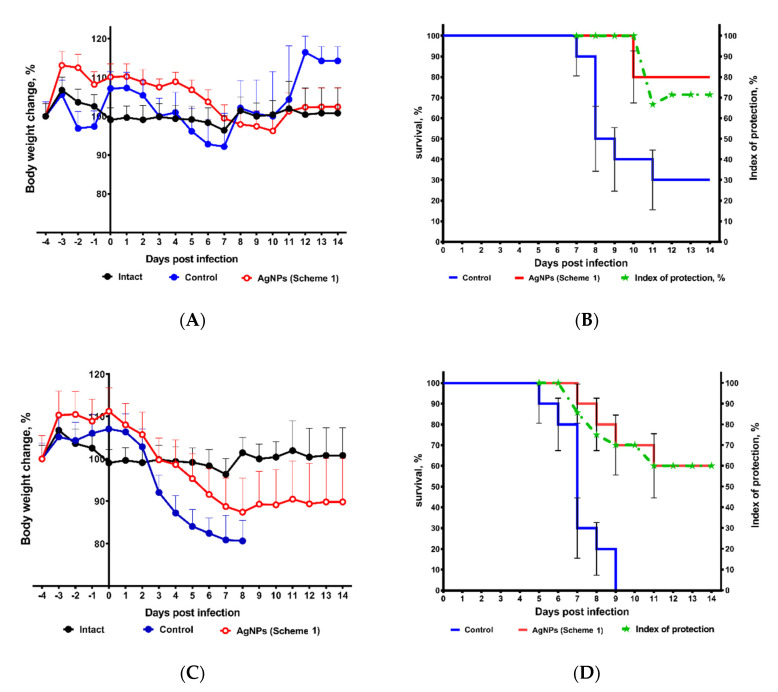
Protection of mice from lethal primary viral pneumonia by AgNPs. Mean weight loss ± SEM (*p* < 0.05) (**A**,**C**) and survival, % ± SE (*p* < 0.05) (**B**,**D**), were monitored daily for 14 days. Mice were inoculated intranasally with 1 LD_50_ (**A**,**B**) or 10 LD_50_ (**C**,**D**).

**Body weight loss and mortality in mice treated by AgNPs according to Scheme 1.** AgNPs inhibited the infection-induced decrease in body weight when used according to Scheme 1 (Figure 6A,C). A slight short-term increase in the body weight of animals treated by AgNPs during the first five days of experiment was unexpectedly detected compared to mice of other groups (Figure 6A,C). In the groups infected with 1 LD_50_ there was no statistical difference in body weight loss, but when the infection dose was increased to 10 LD_50_ there was a difference between the three groups.

In the groups treated by AgNPs, the onset of mortality was delayed by 4 and 2 days compared to the control group for infection doses 1 and 10 LD_50_, respectively. The total survival rate reached 80% and 60%, respectively (Figure 6B,D).

**Protection index.** The calculated dynamic protection index at the virus doses of 1 and 10 LD_50_ varied from 100% to 60–80% and remained at a stable high level of 60–80% until the end of the observation (day 14) (Figure 6B,D). As can be seen from Figure 6B,D, the survival rate of mice treated with AgNPs progressively decreased with increased infectious dose. The highest survival rate of treated mice was observed at a dose of 1 LD_50_ (Figure 6B).

**Figure 7:**
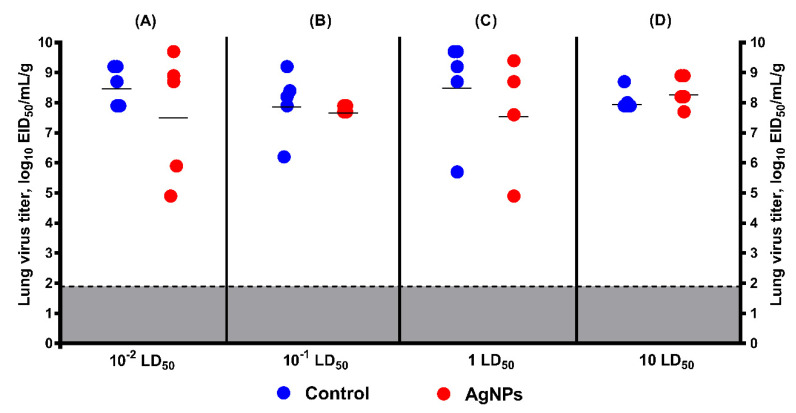
Virus replication in the lungs of mice on D3 post-infection with 10^−2^, 10^−1^, 1, and 10 LD_50_ as measured by titration in chicken embryos (mean ± SD, *p* > 0.05). Mice were infected with the dose of (**A**) 10^−2^ LD_50_; (**B**) 10^−1^ LD_50_; (**C**) 1 LD_50_; and (**D**) 10 LD_50_; red circles—virus titer in lung tissue of control mice; blue circles—virus titer in lung tissue of mice treated by AgNPs according to Scheme 1; gray—the limit of virus detection (1.9 log_10_ EID_50_/mL/g).

**Virus replication in the lower respiratory tract of mice.** The SA virus efficiently multiplied in the lungs of control mice. This was estimated by measuring virus titer in lung tissue samples taken on day 3 post-infection. Virus titers of 8.6 ± 1.6 and 8.1 ± 0.4 log_10_ EID_50_/mL/g of lung tissue were observed in control mice inoculated with 1 and 10 LD_50_, respectively (Figure 7).

In mice inoculated with 1 LD_50_ SA and treated with AgNPs, the average viral lung titer was lower than in control mice (Figure 7C). However, a statistically significant difference was not found (8.6 ± 1.6 log_10_ EID_50_/mL/g of lung tissue in control group versus 7.7 ± 1.9 log_10_ EID_50_/mL/g of lung tissue in treated group). When the infection dose was increased to 10 LD_50_, this difference in the lung virus titers was not clear (Figure 7D), despite the substantial increase in average survival time and decrease in mortality in the treated group compared with the control animals.

For a more comprehensive understanding of the potential effect of AgNPs on virus replication, two more groups were added (10^−1^ LD_50_ and 10^−2^ LD_50_) (Figure 7A,B). In these groups, a statistically significant difference was also not found (8.6 ± 0.7 log_10_ EID_50_/mL/g of lung tissue in control group inoculated with 10^−2^ LD_50_ versus 7.6 ± 2.1 log_10_ EID_50_/mL/g of lung tissue in treated group, and 8 ± 1.1 log_10_ EID_50_/mL/g of lung tissue in control group inoculated with 10^−1^ LD_50_ versus 7.8 ± 0.1 log_10_ EID_50_/mL/g of lung tissue in treated group, respectively).

## 4. Discussion

In this study, we evaluated the effect of the treatment with AgNPs on the influenza virus infection in CBA mice. The AgNP concentration for the treatment of mice was selected so that it was able to significantly lower copper status indexes, while remaining safe for health. It was shown that in Balb/C mice, daily intraperitoneal injections of spherical AgNPs with an average diameter of 20 nm for 9 days at a concentration of 2.5 mg/kg body weight did not cause pathological changes in the morphology of the brain, lungs, heart, and internal organs, with the exception of testicular tissues. Furthermore, in the treated mice, the level of liver enzymes and renal markers in serum did not change [63]. In mice of the same line, a daily injection of 0.4 mg of AgNPs per 1 kg body weight decreased oxidase activity of blood serum by 70% after 3 days [64]. Thus, in this work, mice were treated intraperitoneally with 0.2 mg of AgNPs per 1 kg body mass per day, following the schedules given in Figure 1. The equivalent silver dose was well below the toxicity threshold for mammalian cells [65].

In control mice, after 3 days of infection, the oxidase activity increased approximately twofold. The results are in good agreement with data that show, during influenza infection, the holo-Cp level in the blood increases together with other acute phase proteins and remains high throughout the infection [37,66]. This fact indicates that the increase in Cp gene expression occurs at the transcriptional level. However, the mechanism of the influenza virus action has not been exactly established. It is known that during influenza infection, the level of pro-inflammatory cytokines, including interleukins 1 and 6, increases [67,68,69]. Both upregulate Cp gene activity at the transcription level [70,71].

Treatment of mice with AgNPs for 4 and 7 days leads to a decrease in the holo-Cp level by factors of ~2 and ~6, respectively. As we have shown earlier, the AgNP-mediated decrease in Cp oxidase activity occurred because silver atoms displaced copper ions in the Cp active centers during Cp folding in the Golgi complex lumen, and Cp oxidase activity decreased. However, the concentration of Cp-mRNA, the Cp polypeptide level in the Golgi complex, in addition to the secretion rate and half-life of Ag-Cp, did not change [47]. Thus, AgNPs do not affect any of the levels of Cp gene expression. Therefore, AgNPs and the influenza virus influence copper status in two different ways: AgNPs inhibit the formation of Cp active centers and decrease the holo-Cp level, and the influenza virus induces the Cp gene at the level of transcription and promotes the increase in the holo-Cp level.

In the study, we used the standard testing of Ag-mice infected with the influenza virus for lethality and influenza virus infectivity [72,73]. It was convincingly shown that AgNPs reduce the lethality in mice depending on the treatment regimen with AgNPs and the infectious dose of the influenza virus. Furthermore, virus replication in the lungs did not change significantly.

In mice treated by AgNPs according to Scheme 1, the progression of infection occurred against the background of low oxidase activity. On day 3 post-infection, the oxidase activity in Ag-mice was several times lower than that in mice that were not treated by AgNPs. In the infected Ag-mice, the oxidase activity was clearly higher than in the uninfected mice. The treated mice demonstrated significantly lower mortality and prolonged lifespan compared to untreated infected animals, and the protection index reached 60–70% at the end of the experiment. In addition, the treatment of the animals by AgNPs resulted in the normalization of the weight dynamics of the animals, similar to the results described for oseltamivir (Tamiflu), the most common anti-influenza drug [74]. Moreover, the number of lung lesions per mouse on day 3 decreased by a factor of 6 at a 1 LD_50_ infection dose. However, at a 10 LD_50_ infection dose, the treated group was undistinguishable from the control by this parameter.

Because oxidase activity is restored to normal levels for 3 days following the last AgNPs injection in Ag-mice [44], it could be considered that, in Ag-mice treated according to Scheme 2, the infection developed against the background of growing oxidase activity. When mice were infected with 10 LD_50_, the oxidase activity even exceeded the levels of intact mice. With simultaneous onset of the infection and the administration of AgNPs (Scheme 3), influenza infection developed against the background of a decrease in holo-Cp level induced by AgNPs and an increase in Cp gene activity stimulated by influenza infection. With both Schemes 2 and 3, AgNPs protected mice from developing flu symptoms significantly less efficiently. Our results generally correspond to the data of Xiang et al. who administered AgNPs to mice intranasally for a total of three times post infection at concentrations of 5 mg/kg or 20 mg/kg [55].

The presented data suggest that the effectiveness of AgNP protection correlates with lower elevation of oxidase activity induced by the influenza virus. Thus, the question arises of how AgNPs inhibit the virus-mediated elevation of oxidase activity. No reliable data are available at present to answer this question. However, indirect evidence exists. First, influenza infection causes an increase in the level of neutrophils in the lungs [75], the apoptosis of which leads to an increase in the concentration of pro-inflammatory interleukins [69,76]. Second, holo-Cp induces apoptosis of neutrophils [36]. These data allow us to speculate that, in Ag-mice, the holo-Cp level decreases while Cp protein concentration does not change [47], and the ratio of holo-Cp/apo-Cp is strongly biased towards apo-Cp, which cannot induce apoptosis of neutrophils [42]. It is possible that the high level of Ag-Cp prevents both neutrophil apoptosis and induction of the activity of its own gene. This assumption needs more research.

**Figure 8:**
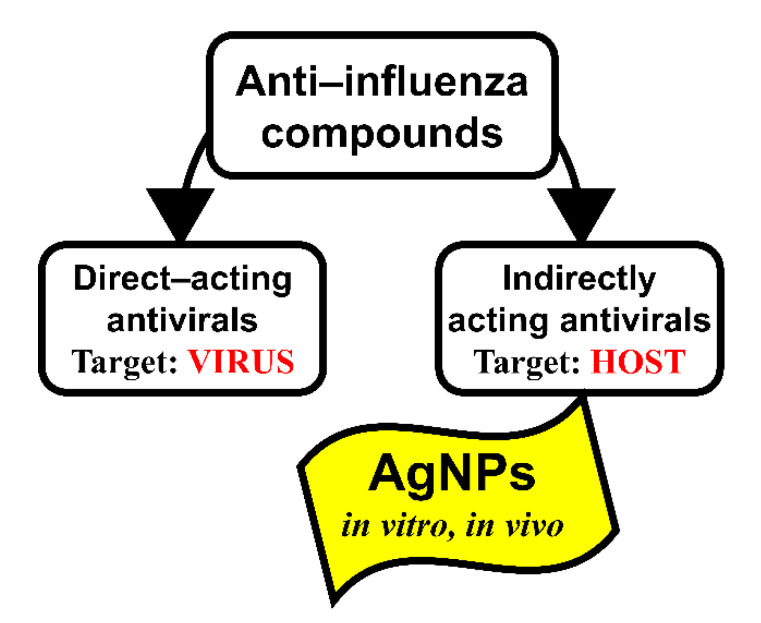
Target-dependent types of anti-influenza virus compounds.

All antivirals including anti-influenza drugs can be classified into two main target-dependent categories: directly acting antivirals (DAAs) and indirectly acting antivirals (IAAs) (Figure 8).

DAAs act on the different viral proteins or stages of the viral life cycle. IAAs act as symptomatic and etiotropic drugs (for instance, pain relievers, fever reducers, vitamins, etc.) or through a change of the metabolism of the host cell that was modified under the influence of a viral infection. When using DAAs, the correlation between the effectiveness of inhibition of the influenza virus replication in the animals’ lungs and the level of reduction of mortality is pronounced [77,78]. However, when using IAAs, the lethality does not always depend on the level of influenza virus replication in vivo. In articles devoted to the effect of nanoparticles on the outcome of influenza infection, there is no direct evidence of their effect on the virus replication [52,53,54]. This can probably explain why in our experiments the absence of pronounced inhibition of virus reproduction was accompanied by a significant decrease in mortality in the groups of mice treated with AgNPs. It is possible that inhibition of the reproduction of the influenza virus in the lower respiratory tract of mice treated with AgNPs is not the mechanism of their anti-influenza action.

All currently licensed influenza drugs belong to the group of DAAs [79]. When compared to indirectly acting host-directed experimental antivirals, DAAs have a lower tendency for undesirable side effects. However, acquired resistance of viruses to DAA drugs is a substantial challenge in fields such as clinical practice and virology. From this perspective, screening of IAAs that are independent of the genetic variability and biological properties of the target virus is of importance. The inhibitory effects of nanosilver on the influenza virus in in vitro and in vivo experiments have been shown in a limited number of studies [53,54,55]. Our results demonstrate that AgNPs as an IAA can be a viable tool for the development of a formulation that efficiently combats any influenza infection.

## 5. Conclusions

In this study, an unparalleled approach to control influenza was developed, based on the restriction of virus infection by shifting the copper balance in the host body using treatment with silver nanoparticles. It is of considerable interest and significance that mice treated with AgNPs were more resistant to infection than control mice. With a continuous presence of AgNPs, significant inhibition and amelioration of H1N1pdm09 influenza infection was observed. Silver nanoparticles administered intraperitoneally at a daily dose of 0.2 mg/kg body weight significantly enhanced survival in mice infected with lethal doses of the H1N1pdm09 influenza virus, prevented the development of pathologic lung lesions, and increased life expectancy. A clear dependence of effects on infection dose and time schedule of AgNP application was noted. This study provides support for the view that silver nanoparticles could be used as protection against influenza.

## Figures and Tables

**Table 1:** Atomic copper and silver concentration in mice infected with the influenza virus and treated with AgNPs.

Mouse Group	Metal Concentration, µM			
	3 Days After Infection		At the End of Experiment	
	[Cu]	[Ag]	[Cu]	[Ag]
Intact	9.4 ± 0.47	0	-	-
AgNP treatment during				
4 days	7.3 ± 0.24	1.9 ± 0.01	-	-
7 days	4.7 ± 0.19	2.8 ± 0.11	-	-
The viral dose of 1 LD_50_ per mouse				
Control	14.6 ± 0.53	-	-	-
Scheme 1	6.5 ± 0.21	1.2 ± 0.03	9.3	3.7
Scheme 2	12.4 ± 0.56	1.3 ± 0.06	12.7	0.25
Scheme 3	12.8 ± 0.39	1.9 ± 0.03	8.8	3.2
The viral dose of 10 LD_50_ per mouse				
control	15.3 ± 0.33	-	-	-
Scheme 1	7.4 ± 0.69	0.9 ± 0.02	8.3	2.3
Scheme 2	12.8 ± 0.53	1.3 ± 0.05	-	-
Scheme 3	13.4 ± 0.17	1.8 ± 0.04	8.5	2.3

**Table 2:** Protective activity of AgNPs in mice infected with lethal doses of SA influenza virus.

Scheme	A Number of Fatal Cases on Day Post-Infection (*n* = 10)											Lethal Cases ^1^	Lung Lesions ^2^	ALE ^3^, Days	IP ^4^
	4	5	6	7	8	9	10	11	12	13	14				
The viral dose of 1 LD_50_ per mouse															
Control	0	0	1	3	1	0	1	1	0	0	0	70%	40.0%	9.8	NA ^5^
Scheme 1	0	0	0	0	0	0	2	0	0	0	0	20%	6.3%	13.2	71%
Scheme 2	0	0	1	2	0	2	0	0	0	1	0	60%	60.0%	10.7	14%
Scheme 3	0	0	1	1	3	1	1	0	0	0	0	70%	ND ^6^	9.8	0%
The viral dose of 10 LD_50_ per mouse															
Control	1	1	5	0	3	0	0	0	0	0	0	100%	75.0%	6.2	NA
Scheme 1	0	0	1	1	1	0	1	0	0	0	0	40%	25.0%	11.5	60%
Scheme 2	0	1	0	3	2	2	1	1	0	0	0	100%	65.0%	8.1	0%
Scheme 3	0	0	2	2	2	0	0	2	0	0	0	80%	ND	9.2	20%

^1^ Lethality on D14. ^2^ Mean lung lesions per mouse on D3. ^3^ Average life expectancy. ^4^ Index of protection on D14. ^5^ NA—not applicable. ^6^ ND—not determined.
